# The Efficacy of 100 and 300 mg Gabapentin in the Treatment of Carpal Tunnel Syndrome

**Published:** 2015

**Authors:** Bina Eftekharsadat, Arash Babaei-Ghazani, Afshin Habibzadeh

**Affiliations:** a *Physical Medicine and Rehabilitation Research Center, Tabriz University of Medical Sciences, Tabriz, Iran. *; b *Department of Physical Medicine and Rehabilitation, Iran University of Medical Sciences, Tehran, Iran. *; c *Medical Philosophy and History Research Center, Tabriz University of Medical Sciences, Tabriz, Iran.*

**Keywords:** Gabapentin, Carpal tunnel syndrome, BCTQ, VAS

## Abstract

Carpal tunnel syndrome (CTS) is a neuropathy due to the compression of the median nerve. It is shown that gabapentin in high doses is effective in treatment of CTS patients. In this study we evaluated the efficacy of low doses of gabapentin in treatment of CTS patients. Ninety patients with CTS were randomly assigned to groups A, B and C. Gabapentin was administered to group A with dose of 100 mg/day and to group B with dose of 300 mg/day for 2 months. Group C received no treatment. Before and after treatment, patients were evaluated using Visual analogue scale (VAS) for pain and parasthesia, Boston carpal tunnel questionnaire (BCTQ) including Symptom Severity Scale (SSS) and Functional Status Scale (FSS) to evaluate the efficacy of the treatment. The pinch and grip strength was also measured. There was significant improvement in VAS, grip strength, pinch strength, SSS, FSS and BCTQ score in all three groups (p < 0.05), but the changes in CMAP and SNAP was not significant. Groups A and B in comparison to group C had significantly better improvement in VAS, pinch strength, SSS, FSS and BCTQ total score (p < 0.05). There was significantly more improvement in pinch strength and SSS score in group B compared to group A (p < 0.05). Gabapentin in low doses is a useful drug in treatment of CTS symptoms with no side effects and intolerance. Gabapentin with dose of 300 mg/day is more effective than the dose of 100 mg/day.

## Introduction

Carpal tunnel syndrome (CTS) is one of the most common entrapment neuropathy which results from the compression of the median nerve at the carpal tunnel level in the wrist (1, 2). CTS symptoms are paresthesia (which is commonly exacerbated at night), numbness, tingling, pain (more often nocturnal) and weakness (1-4). Diagnosis is based on clinical presentation and confirmed by electrodiagnostic studies (2, 5).

It is critical to begin treating early phases of carpal tunnel syndrome before the damage progresses. Initial therapies for CTS include oral or local injection of corticosteroids, NSAIDS, splinting and activity modification (6). The anticonvulsants such as pregabalin and gabapentin are proposed for the treatment of neuropathic pain (7-10). Gabapentin use is shown to be safe and effective in CTS (as a neuropathy) (11-14). These studies have used gabapentin with high doses (900-1800 mg/day) which would have some side effects and is not tolerable by some patients. There is a possibility that gabapentin reach satisfactory results with low doses. To evaluate this presumption, we aim to evaluate the efficacy of 100 mg/day and 300 mg/day gabapentin in the symptom alleviation in patients with CTS.

## Experimental

*Methods*

In this randomized clinical trial we studied 90 consecutive patients with mild to moderate CTS (70 women and 20 men, mean age 44.38 ± 8.26 years, range, 22-68 years) visiting Physical Medicine and Rehabilitation clinics, Tabriz University of Medical sciences, Tabriz, Iran. Clinical diagnosis of CTS was based on the American Academy of Neurology diagnostic criteria (15). Comprehensive medical and neurological evaluations were performed to exclude neuropathies of other etiologies. Patients with isolated CTS were included in the study and patients with CTS in the setting of other neurological diseases were excluded. Patients who had thenar atrophy or were treated with steroid injection in the last 3 months, with known contraindication to or prior use of any antiepileptic drugs, creatinine clearance <30 mL/min or elevated liver enzymes, clinically significant hematologic, cardiovascular, renal, or hepatic disease, history of narcotic or alcohol abuse and previous CTS surgery were also excluded. The protocol was approved by the local ethics committee of our institution, and the informed consent was obtained from all study participants. This clinical trial was registered in the Iranian Clinical Trial Registry, and the registry number is IRCT201401144641N7.

Using RANDLIST 1.2 software, random numbers were produced and according to sample size, patients were enrolled into the study. In the case of missing or exclusion of any patient during the course of treatment, a new patient was selected by more random numbers. Patients with mild to moderate CTS randomly divided into 3 groups A and B and C. For a period of 2 months, Group A received gabapentin 100 mg/day (Soha Pharmaceutical Co., Iran), Group B received gabapentin 300 mg/day and group C received no treatment. Splints were administered at nights for all 3 groups during the study. The physician evaluating the treatment outcome was blinded to the regimens. The possible side effects were also monitored to assess the safety of the drug. Patients were asked to report or visit the clinic if any adverse effects appeared during the study period. At baseline and at the end of the 2 months treatment period, patients were asked to complete Boston carpal tunnel questionnaire (BCTQ) to evaluate the efficacy of the treatment. The pain and parasthesia was measured with visual analogue scale (VAS). Pinch and grip strength were also measured using dynamometer.

Boston carpal tunnel questionnaire (BCTQ), a self-administered disease-specific outcome instrument, was used to assess the severity of symptoms and the functional status. The questionnaire consists of two multi-item scales: the Symptom Severity Scale (SSS) and the Functional Status Scale (FSS). The SSS evaluates symptoms like pain, numbness, weakness, paraesthesia, or clumsiness and over-all functional status using 11 questions. The FSS evaluates difficulties with daily activities like writing, buttoning clothes, holding a book while reading, gripping a telephone handle, opening jars, household chores, carrying grocery bags, and bathing/ dressing using eight questions. The SSS consisted of responses numbered 1 (no symptoms) to 5 (most severe). The FSS consisted of responses numbered 1 (performance with no difficulty) to 5 (unable to perform due to symptoms). The overall SSS and FSS scores are calculated as the mean of the scores. 

Pain and paresthesia was measured using a 10 cm visual analogue scale (VAS). Pain intensity is referred as 0 to 10, in which 0 = no pain at all and 10 = the worst pain possible. Patients were asked to sign on the VAS scale that corresponded their pain. Grip strength was measured by a Lafayette hand dynamometer and pinch strength was determined by Lafayette Hydraulic Pinch Gauge (3700 Sagamore Parkway North, Lafayette, Indiana, USA).

Electrodiagnostic assessment was carried out using a ‘Medelec TECA synergy’ electromyography device, controlling the skin temperature at 32°C. Electrodiagnostic data, including distal latency and amplitude of ‘compound motor action potential’ (CMAP) and ‘sensory nerve action potential’ (SNAP) of the median nerve were derived from electrodiagnostic study reports and recorded on the patient’s data form. 

*Statistical analysis*

Statistical analyses were performed using the Statistical Package for Social Sciences, version 17.0 (SPSS, Chicago, Illinois). A p value of < 0.05 was considered statistically significant.

## Results and Disscusion

Ninety patients with CTS were evaluated in three therapeutic groups. Table 1 demonstrates baseline findings between groups. There was no significant difference between groups. The initial presenting symptoms of CTS were numbness (95.5%), tingling (91.1%) and nocturnal worsening of symptoms (90%). Phalen's and Tinel's sign were positive in 82.2% and 71.1% of patients, respectively. 

**Table 1:** Patients’ baseline findings

		**Group A**	**Group B**	**Group C**	**P value**
Age (years)		42.46 ± 8.16	44.56 ± 8.58	46.34 ± 8.08	NS
Gender, Female		24 (80%)	21 (70%)	25 (83.3%)	NS
Mean duration of illness		21.56 ± 7.34	20.32 ± 7.93	21.33 ± 7.45	NS
Dominant hand	Right	28 (93.3%)	23 (76.7%)	27 (90%)	NS
	Left	2 (6.7%)	7 (23.3%)	3 (10%)	
Involved hand	Right	18 (60%)	18 (60%)	19 (63.3%)	NS
	Left	12 (40%)	12 (40%)	11 (36.7%)	

NS: Not significant.

The changes in the studied parameters before and after treatment are shown in Table 2. Before the treatment, there were no significant differences between groups while after the treatment there were significant differences in VAS, strength, pinch, SSS, FSS and total BCTQ score between groups. After the treatment, Group A and B in comparison to group C had significantly lower VAS, SSS, FSS and BCTQ total and higher pinch strength (p < 0.05), but the differences between group A and B were not significant. Comparing the results before and after treatment, we observed significant improvement in VAS, grip strength, pinch strength, SSS score, FSS score and BCTQ score in all three groups (p < 0.001 in all variables in each groups); however there were no significant changes in CMAP and SNAP. 

**Table 2:** Changes in the studied parameters before and 2 months after treatment

		**Group A**	**Group B**	**Group C**	**P value**
VAS	Before	5.63±2.02	4.93±2.34	5.70±2.11	NS
	After	1.96±1.66	1.55±1.47	3.37±1.94	<0.001*
Grip Strength	Before	21.94±4.74	22.27±4.53	23.11±3.50	NS
	After	25.38±4.47	25.94±4.26	24.60±3.33	NS
Pinch strength	Before	4.31±0.67	4.27±1.05	4.02±0.65	NS
	After	5.67±0.73	6.08±0.90	4.50±0.85	<0.001*
SSS	Before	3.50±0.43	3.65±0.33	3.48±0.56	NS
	After	2.41±0.52	2.19±0.43	2.86±0.51	<0.001*
FSS	Before	4.32±0.52	4.36±0.54	4.12±0.47	NS
	After	3.22±0.95	3.17±0.72	3.70±0.56	0.02*
BCTQ total	Before	7.83±0.87	8.01±0.83	7.60±0.89	NS
	After	5.70±1.34	5.36±0.98	6.57±0.87	<0.001
CMAP	Before	4.17±0.42	4.98±0.76	4.49±0.42	NS
	After	4.17±0.44	5.78±1.58	4.46±0.41	NS
SNAP	Before	3.49±0.29	4.49±0.95	4.65±1.05	NS
	After	3.46±0.32	4.72±1.20	3.56±0.37	NS

VAS: Visual analogue scale; SSS: Symptom Severity Scale; FSS: Functional Status Scale; BCTQ: Boston carpal tunnel questionnaire; CMAP: Compound motor action potential; SNAP: Sensory nerve action potential; NS: Not significant.

* p is two-sided significant.

In order to evaluate the more effective treatment between groups, we evaluated the percent of improvement in each variable between groups (Table 3). There were significant differences between groups in all evaluated parameters (p < 0.001). Groups A and B in comparison to group C had significantly better improvement in VAS, pinch strength, SSS, FSS and BCTQ total (p < 0.001). Comparing the results between group A and B, there were significantly more improvement in pinch strength and SSS score group B (p < 0.05), but the differences between groups in other variables were not significant.

We observed no side effects during the study period in any of the subjects and all subjects had a good compliance to the treatment. 

**Table 3:** Rate of improvement in studied variables between groups

	**Group A**	**Group B**	**Group C**	**P value**
VAS	-70.08±20.72%	-75.57±20.79	-45.78±19.60	<0.001*
Grip strength	17.25±12.49%	17.51±8.95%	6.84±4.74%	<0.001*
Pinch strength	33.30±18.67%	49.81±28.17%	15.43±4.60%	<0.001*
SSS	-30.74±14.23	-39.46±13.67	-16.62±13.86	<0.001*
FSS	-24.63±17.91	-27.16±15.33	-9.71±12.46	<0.001*
BCTQ total	-27.45±14.07	-32.78±12.35	-13.12±9.97	<0.001*

VAS: Visual analogue scale; SSS: Symptom Severity Scale; FSS: Functional Status Scale; BCTQ: Boston carpal tunnel questionnaire

* p is two-sided significant.

Gabapentin is an effective drug for treatment of neuropathic pain and has been reported to be effective in various disease including trigeminal neuralgia, postherpetic neuralgias, diabetic neuropathy and after surgeries especially orthopedic and neurosurgery (9, 16, 17). The drug’s effect in improving neuropathic pain has encouraged many others to use gabapentin in other diseases with neuropathy basis including CTS (11-14).

In this prospective randomized clinical trial we evaluated the effects of low dose gabapentin (100 mg and 300 mg) in controlling pain and improving symptoms of patients with CTS. Following treatment with gabapentin 100 and 300 mg per day, patients reported significant decrease in pain severity and BCTQ subscores (p < 0.001). There was also a significant increase in pinch and grip strength (p < 0.001).

There are few studies evaluating gabapentin effects on relieving CTS symptoms which are performed with high doses of gabapentin (11-14). Taverner *et al*. (11) reported significant improvement in signs and symptoms of CTS in 84.2% of their patients (n = 19) with gabapentin 1800 m/day during 6 months follow-up. Duman *et al.* (12) reported a reduction in symptoms in 21 patients with CTS patients treated for three months using gabapentin with average dose of 648 mg/day. Erdemoglu (13) in a study of 41 patients with CTS reported significant decrease in SSS and FSS and total BCTQ during follow-up using 1800 mg/day gabapentin. However, all these three previous studies were single group study with no control group and randomization. The recent randomized clinical trial by Hui and colleagues (14) also showed significant differences between gabapentin (300-900 mg/day) and placebo group in 2 weeks, but by eight weeks they observed no significant improvement (14). In our study at the end of eight week trial we observed significantly better improvement in patients receiving gabapentin 100 or 300 mg in comparison to control group (p < 0.001).

The recommended and tolerable dosage of gabapentin in the literature is reported between 900 and 3600 mg per day (11). However the higher doses would have some adverse effects and not well tolerated by all patients which can cause treatment failure. Taverner *et al*. (11) reported that 28% of patients stopped gabapentin because of side effects. Erdemoglu (13) also reported side effects of high dose gabapentin in 26.8% of CTS patients which was mild and tolerable. Unlike previous studies which used Gabapentin between 600-1800 mg/day (11-14), we only administered low doses of gabapentin (100 and 300 mg/day) which yielded similar beneficiary results. Patients reported no side effects and complications during gabapentin treatment and the drug was well tolerated. It is reported that symptom relief occurs during the titration period even before the maximal dose is achieved. In other studies the lower doses of gabapentin has also shown to be effective with low side effects and intolerance (17-22). Panah Khahi and colleagues (17) reported significant improvement in pain score following 300 mg of gabapentin administration before orthopedic surgery. These results are indicative of beneficiary results of low dose gabapentin, which accompanies with lower side effects, higher tolerance as well as lower costs of treatment.

Among previous studies, only one had a control group (14) and the others have evaluated the results only in one group. It is possible that other factors have role in treatment improvement beside gabapentin. Having a control group would allow researchers to evaluate the effects of other parameters beside the drug on the final outcome. We observed significant improvement in all three groups, which means that using splints at nights alone can significantly alter the patients’ situation. However, observing significantly more improvement in gabapentin groups compared to control group is indicative of efficacy of treatment with gabapentin. Unlike our findings, Hui and colleagues (14) having a placebo group found no difference in the mean reduction of symptom severity between gabapentin and placebo group at the end of the study. 

We also evaluated the results of two different doses of gabapentin, 100 and 300 mg daily and observed that despite significant improvements in all measured parameters in both groups, patients receiving gabapentin 300 mg/day showed better pinch strength and SSS score. As there were no side effects for any of the gabapentin doses, it could be recommended to use 300 mg/day for more beneficiary results.

## Conclusion

In conclusion, gabapentin is a useful drug in treatment of CTS symptoms which can be used with low doses and have no side effects and intolerance. Using gabapentin with dose of 300 mg/day in comparison to 100 mg/day is recommended because of more satisfactory results during the treatment. 

## References

[1] Aroori S, Spence RA (2008). Carpal tunnel syndrome. Ulster. Med. J..

[2] Eftekharsadat B, Kazem Shakouri S, Shimia M, Rahbar M, Ghojazadeh M, Reza Rashidi M, Hadi Faraji M (2011). Effect of E. laciniata (L) ointment on mild and moderate carpal tunnel syndrome: a double-blind, randomized clinical trial. Phytother. Res..

[3] Bland JD, Weller P, Rudolfer S (2011). Questionnaire tools for the diagnosis of carpal tunnel syndrome from the patient history. Muscle. Nerve..

[4] Sharifi-Mollayousefi A, Yazdchi-Marandi M, Ayramlou H, Heidari P, Salavati A, Zarrintan S, Sharifi-Mollayousefi A (Warsz). Assessment of body mass index and hand anthropometric measurements as independent risk factors for carpal tunnel syndrome. Folia. Morphol..

[5] Keith MW, Masear V, Chung K, Maupin K, Andary M, Amadio PC, Barth RW, Watters WC 3rd, Goldberg MJ, Haralson RH 3rd, Turkelson CM, Wies JL (2009). Diagnosis of carpal tunnel syndrome. J. Am. Acad. Orthop. Surg..

[6] Talebi M, Andalib S, Bakhti S, Ayromlou H, Aghili A, Talebi A (2013). Effect of vitamin b6 on clinical symptoms and electrodiagnostic results of patients with carpal tunnel syndrome. Adv. Pharm. Bull..

[7] Freynhagen R, Strojek K, Griesing T, Whalen E, Balkenohl M (2005). Efficacy of pregabalin in neuropathic pain evaluated in a 12–weeks, randomised, double–blind, multicentre, placebo-controlled trial of flexible- and fixed dose regimens. Pain..

[8] Strojek K, Flöter T, Balkenohl M, Xie F, Griesing T (2004). Pregabalin in the management of chronic neuropathic pain (NeP): a novel evaluation of flexible and fixed dosing. J. Pain..

[9] Backonja M, Beydoun A, Edwards KR, Schwartz SL, Fonseca V, Hes M, LaMoreaux L, Garofalo E (1998). Gabapentin for the symptomatic treatment of painful neuropathy in patients with diabetes mellitus: a randomized controlled study. JAMA..

[10] Rowbotham M, Harden N, Stacey B, Bernstein P, Magnus-Miller L (1998). Gabapentin in the treatment of post-herpetic neuralgia: a randomized controlled trial. JAMA..

[11] Taverner D, Lisbona MP, Segalés N, Docampo E, Calvet J, Castro S, Benito P (Barc). Efficacy of gabapentin in the treatment of carpal tunnel syndrome. Med. Clin..

[12] Duman I, Aydemir K, Ozgul A, Kalyon TA (2008). Assessment of the efficacy of gabapentin in carpal tunnel syndrome. J. Clin. Rheumatol..

[13] Erdemoglu AK (2009). The efficacy and safety of gabapentin in carpal tunnel patients: open label trial. Neurol. India..

[14] Hui AC, Wong SM, Leung HW, Man BL, Yu E, Wong LK (2011). Gabapentin for the treatment of carpal tunnel syndrome: a randomized controlled trial. Eur. J. Neurol..

[15] (1993). Practice parameter for carpal tunnel syndrome (summary statement): Report of the Quality Standards Subcommittee of the American Academy of Neurology. Neurology.

[16] Coderre TJ, Kumar N, Lefebvre CD, Yu JS (2005). Evidence that gabapentin reduces neuropathic pain by inhibiting the spinal release of glutamate. J. Neurochem..

[17] Panah Khahi M, Yaghooti AA, Marashi SH, Nadjafi A (2011). Effect of pre-emptive gabapentin on postoperative pain following lower extremity orthopaedic surgery under spinal anaesthesia. Singapore. Med. J..

[18] Turan A, White PF, Karamanlioglu B, Pamukçu Z (2007). Premedication with gabapentin: the effect on tourniquet pain and quality of intravenous regional anesthesia. Anesth. Analg..

[19] Pandey CK, Sahay S, Gupta D, Ambesh SP, Singh RB, Raza M, Singh U, Singh PK (2004). Preemptive gabapentin decreases postoperative pain after lumbar discoidectomy. Can. J. Anaesth..

[20] Pandey CK, Priye S, Singh S, Singh U, Singh RB, Singh PK (2004). Preemptive use of gabapentin significantly decreases postoperative pain and rescue analgesic requirements in laparoscopic cholecystectomy. Can. J. Anaesth..

[21] Ahmadi M, Samadbeik M, Sadoughi F (2014). Modeling of outpatient prescribing process in iran: a gateway toward electronic prescribing system. Iran J Pharm Res..

[22] Davoodi SH, Hajimiresmaiel SJ, Ajami M, Mohseni-Bandpei A, Ayatollahi SA, Dowlatshahi K, Javedan G, Pazoki-Toroudi H (2014). Caffeine treatment prevented from weight regain after calorie shifting diet induced weight loss. Iran J Pharm Res..

